# Surface complexation modeling of Cu(II) adsorption on mixtures of hydrous ferric oxide and kaolinite

**DOI:** 10.1186/1467-4866-9-9

**Published:** 2008-09-10

**Authors:** Tracy J Lund, Carla M Koretsky, Christopher J Landry, Melinda S Schaller, Soumya Das

**Affiliations:** 1 Arizona State University, School of Earth and Space Exploration, USA; 2 Department of Geosciences, Western Michigan University, Kalamazoo, MI 49008 USA; 3 Pennsylvania State University, Department of Energy and Mineral Engineering, USA; 4 Rutgers, The State University of New Jersey, Department of Environmental Sciences, USA

## Abstract

**Background:**

The application of surface complexation models (SCMs) to natural sediments and soils is hindered by a lack of consistent models and data for large suites of metals and minerals of interest. Furthermore, the surface complexation approach has mostly been developed and tested for single solid systems. Few studies have extended the SCM approach to systems containing multiple solids.

**Results:**

Cu adsorption was measured on pure hydrous ferric oxide (HFO), pure kaolinite (from two sources) and in systems containing mixtures of HFO and kaolinite over a wide range of pH, ionic strength, sorbate/sorbent ratios and, for the mixed solid systems, using a range of kaolinite/HFO ratios. Cu adsorption data measured for the HFO and kaolinite systems was used to derive diffuse layer surface complexation models (DLMs) describing Cu adsorption. Cu adsorption on HFO is reasonably well described using a 1-site or 2-site DLM. Adsorption of Cu on kaolinite could be described using a simple 1-site DLM with formation of a monodentate Cu complex on a variable charge surface site. However, for consistency with models derived for weaker sorbing cations, a 2-site DLM with a variable charge and a permanent charge site was also developed.

**Conclusion:**

Component additivity predictions of speciation in mixed mineral systems based on DLM parameters derived for the pure mineral systems were in good agreement with measured data. Discrepancies between the model predictions and measured data were similar to those observed for the calibrated pure mineral systems. The results suggest that quantifying specific interactions between HFO and kaolinite in speciation models may not be necessary. However, before the component additivity approach can be applied to natural sediments and soils, the effects of aging must be further studied and methods must be developed to estimate reactive surface areas of solid constituents in natural samples.

## Background

Metal speciation is a primary control on metal mobility and bioavailability in the environment, and adsorption reactions can play a significant role in this process (e.g., [1]). Therefore, many researchers have worked to develop predictive models to describe metal adsorption for a wide range of systems. In studies of natural sediments and soils, empirical approaches based on partition coefficients (K_d_) or semi-empirical Langmuir or Freundlich isotherms are often used to describe metal partitioning between solutions and solid substrates. However, because partition coefficients depend on solution and substrate composition, they cannot be extrapolated beyond the conditions for which they are measured (e.g., [2,3]). Furthermore, because partition coefficients do not include any consideration of mass balance, they can result in very misleading predictions regarding metal speciation and mobility [2]. The application of Langmuir or Freundlich isotherms is similarly hindered because these also depend on solution and substrate composition and do not account for the development of electrical charge at mineral surfaces, nor do they consider the structure of adsorbed species [3]. In contrast, thermodynamically-based surface complexation models (SCMs) include explicit descriptions of reaction stoichiometries and the development of electrical charge at the solid surface [4]. These models have a significant advantage over empirical or semi-empirical models because once calibrated, they should allow accurate prediction of metal speciation under varying solution compositions (e.g. in ionic strength, background electrolyte, competing ions, etc.), and thus should be useful in predicting metal speciation in a wide variety of systems.

In spite of the significant potential advantages of surface complexation models (SCMs), widespread application of these models, especially to complex sediments and soils, has been lacking for a variety of reasons. Determining the mineralogy of the finest, and therefore highest surface area and presumably most reactive, constituents of soils and sediments is often difficult. Even when the bulk mineralogy is well characterized, deriving reactive surface areas to include in SCMs can be hampered by a lack of information regarding flow paths and the presence of coatings at solid surfaces (e.g., [3,5]). Furthermore, there is a lack of data for adsorption of many metals on substrates that are relevant for natural systems. Lastly, there is little information regarding the applicability of surface models parameterized using pure, single solid systems to more complex systems containing mixtures of solids (e.g. [6,7]). Overcoming these obstacles is crucial if existing surface complexation models are to become widely used and useful for understanding metal speciation in natural systems.

Developing models which are better able to accurately predict the speciation of copper in the environment is important because, although ecosystems require trace quantities of copper to maintain physiological functions [8], at higher concentrations copper is toxic to both plants and animals [9]. Furthermore, copper tends to bind strongly to organic and mineral substrates, potentially resulting in mobilization of competing metal ions. Concentrations of trace metals, including copper, have increased dramatically in many ecosystems worldwide due to anthropogenic activities, including dredging of river sediments [10-13], application of pesticides and fungicides [14,15], and through mining and smelting operations. This has resulted in toxic levels of trace metals in many soils and sediments [8,9,16], and a pressing need to develop accurate predictive models of Cu speciation in the environment.

In order to better understand and quantify copper bioavailability and transport, copper adsorption has been extensively studied. However, Cu adsorption has been described using SCMs for a relatively small suite of single, pure minerals (e.g. Cu/goethite: [17-20]; Cu/hematite: [21]; Cu/gibbsite: [22]; Cu/kaolinite: [23-28]; Cu/hydrous manganese oxide: [29]; Cu/hydrous ferric oxide: [4]) and in even fewer studies in the presence of mixed mineral assemblages (e.g., [6]) or natural soils or sediments (e.g., [30,31]). Furthermore, many of the existing SCMs cannot be used to model adsorption of copper on mixtures of minerals, because the surface complexation parameters have been derived using different treatments of the electrical double layer (e.g. constant capacitance, double layer, triple layer models).

The goal of this study is to develop internally-consistent descriptions of copper adsorption on hydrous ferric oxide and kaolinite at a variety of ionic strength and sorbate/sorbent ratios using a diffuse double layer model (DLM). Models for the individual solid systems are assessed using 95% confidence intervals of a goodness-of-fit parameter, V(Y). The performance of DLMs parameterized using single solid systems are assessed in mixture solid systems by quantitative comparison of measurements and predictions based on V(Y). The double layer model is chosen because of the extensive database of stability constants that has been derived for metal adsorption on hydrous ferric oxide [4] and hydrous manganese oxide [29]. Furthermore, the DLM approach requires fewer fit parameters than other SCMs (e.g. triple layer models) and yields stability constants that, at least in theory, do not vary with ionic strength (unlike those obtained using a constant capacitance model). These features make the DLM approach an attractive option for modeling adsorption on natural sediments and soils.

## Experimental methods

### Materials

All reagents used were ACS reagent or trace metal grade. DDI water was purified using a Barnstead E-pure (Model D4641) water system. Powdered kaolinite from Edgar, Florida was purchased from Ward's Scientific. X-ray diffraction indicates that the kaolinite is moderately well ordered with quartz and mica or smectite impurities, with perhaps 1–2% mica in the < 1 μm size fraction (data courtesy of Ray Ferrell, Louisiana State University). Powdered low defect kaolinite from Washington County, GA, USA (KGa-1b) was obtained from the Clay Minerals Society Source Clays Repository. The most significant impurities in the KGa-1b kaolinite are ~1.64% TiO_2 _[32,33], 0.21% Fe_2_O_3 _[32] and 231 ppm total organic carbon [32]. Kaolinite powder was dried at 90°C for 4 days, and then stored in a refrigerator until usage. No other precleaning was done. Hydrous ferric oxide (HFO) was synthesized based on procedures proposed by Schwertmann and Cornell [34]. Briefly, ~40 g of ferric nitrate was dissolved in ~500 mL of DDI water in a glass beaker. Concentrated trace metal grade NaOH was slowly titrated into the beaker under constant stirring as precipitate formed, until the mixture reached a pH of ~7.0. The mixture was kept at pH 7 for ~72 hours, then poured into a plastic tube, centrifuged, the supernatant decanted, and the remaining precipitate washed with DDI. The centrifuging and washing procedure was repeated ~5–6 times. The final washed precipitate was freeze-dried and the freeze-dried solid ground gently using a mortar and pestle to break up large aggregates.

Specific surface areas for HFO and both types of kaolinite were determined at atmospheric pressure using a Quantachrome Nova Surface and Pore Analyzer Model 2200e. Replicate ~2 g samples of each solid were degassed for ~24 hrs and analyzed using 11-point N_2 _BET. A degassing temperature of ~80°C was used for the HFO and 25°C was used for both types of kaolinite. Measured specific surface areas were: 220 (HFO), 13.6 (KGa-1b kaolinite), and 25.7 (Ward's kaolinite) m^2^/g. Dzombak and Morel [4] argue that due to the presence of significant microporosity N_2 _BET underestimates the surface area available for sorbates on HFO, and therefore suggest that a specific surface area of 600 m^2^/g be adopted for modeling. This recommendation has been widely used in DLM descriptions of metal adsorption on HFO. To be consistent with these previous modeling efforts, this value is adopted here for all surface complexation models derived for Cu adsorption on HFO.

### Experimental approach

Adsorption experiments were completed using continuously stirred batch reactors (500 mL), at room temperature and open to the atmosphere, containing dissolved Cu(II) and NaNO_3 _as the background electrolyte. Batch reactors were typically titrated first to an acidic initial pH (~2–4.5) using trace metal grade HNO_3_. A 10 mL aliquot of this initial suspension of Cu and NaNO_3 _was removed for subsequent analysis of the initial Cu concentration. Next, the HFO, kaolinite or mixture of these solids was added to the well-stirred 500 mL batch reactor. This suspension was typically preequilibrated for 24 hours. The preequilibration procedure may result in some dissolution, especially of kaolinite at low pH. However, Landry et al. [7] demonstrate that preequilibration at acidic compared to circumneutral pH does not significantly influence Co adsorption on kaolinite under conditions similar to those used here. Thus, although some dissolution of the solids may have occurred during preequilibration, this should not significantly influence metal adsorption at the conditions used in this study. After 24 hours, the pH of the preequilibrated suspension was titrated upwards by additions of small volumes of 0.1 M NaOH sufficient to increase the pH by increments of 0.2 to 0.5. Several experiments were also completed in which the base titration was followed by an acid titration using 0.1 M HNO_3_. The acid and base legs of these experiments exhibited no significant hysteresis, i.e. any hysteresis was less than the experimental uncertainty (see also below). After titrant addition and stabilization of the pH to within 0.05 pH log units per minute, which typically occurred in about 10 minutes, a 10 mL aliquot of the mixed suspension was pipetted into an acid-washed 15 mL plastic centrifuge vial. The 15 mL tubes, including the initial mineral-free control sample, were subsequently agitated with a benchtop shaker for 24 hrs, removed from the shaker and the pH measured again. The 24 hr period should be more than sufficient for the adsorption reaction to reach equilibrium (see below). In most experiments, each aliquot was then centrifuged and the supernatant filtered through a 0.2 μm syringe filter. However, several experiments were also completed to compare the effect of syringe-filtering to centrifugation only. No significant difference was observed between samples prepared by filtering and those prepared by centrifugation only. All supernatants were acidified using concentrated trace metal grade HNO_3_, amended to 1000 ppb with an internal indium standard and analyzed for Cu using either a ThermoElectron PQ Excell ICP-MS or a Perkin Elmer Optima 2100DV ICP-OES with matrix-matched calibration standards. The amount of Cu adsorbed was calculated by the difference between Cu concentration in the analyzed supernatants and the initial Cu solution.

To determine the adsorption kinetics and the reversibility of Cu adsorption, adsorption and desorption of Cu on kaolinite was tested as a function of time. Using a batch slurry of 2 g/L KGa kaolinite, 10^-5 ^M Cu and 0.01 M NaNO_3_, adsorption was initiated by titrating the slurry to a pH of 10.5. 98 ± 2% of the initial Cu was adsorbed by the kaolinite within 5 minutes (data not shown). Periodic sampling over the following 72 hours demonstrated that this Cu remained sorbed on the kaolinite surface. To test the reversibility of sorption, after the 72 hour period the slurry was titrated to pH 2.3. Within 10 minutes only 9 ± 4% of the Cu remained sorbed to the kaolinite and after 24 hours 100% of the Cu was recovered from the kaolinite. Under the conditions of the initial sorption experiment (pH 10.5, 10^-5 ^M Cu, 0.01 M NaNO_3_), tenorite is supersaturated and might precipitate. Although it is not possible to distinguish adsorption from surface precipitation in these macroscopic experiments, the rapid desorption of the Cu suggests that adsorption, rather than precipitation, occurs.

### Modeling approach

Surface complexation stability constants for individual adsorption edge experiments were optimized using FITEQL4.0 [35]. Each optimization was completed for a specific reaction stoichiometry (see Table 1 and discussion below) with activity corrections based on the Davies equation (see [35]) and including a stability constant of -7.29 for formation of CuOH^+^_(aq) _from the JCHESS default thermodynamic database, which is based on the EQ3/6 database [36]. Due to their small influence (< 2.5%) on calculated Cu speciation at the measured conditions, CO_2(g) _and other aqueous Cu species were not included in the FITEQL input files. The optimization procedure was used to obtain best-fit stability constants for each edge obtained in a single experiment. In some cases, replicate experiments were completed. The resulting edges were fit individually, and were not aggregated in the modeling. Sets of edges were obtained on kaolinite and HFO to span a range of ionic strength and sorbate/sorbent ratios (see below). The median stability constant(s) derived for sorption onto each solid was input into the speciation code JCHESS, together with all reaction constituents, including CO_2(g)_. The resulting edges were calculated in JCHESS with activities based on the Debye-Huckel equation and using the default JCHESS thermodynamic database, which includes stability constants for formation of HNO_3(aq)_, HCO_3_^-^_(aq)_, CO_2(aq)_, CuOH^+^_(aq)_, CuO_2_^-2^_(aq)_, NaHCO_3_^-^_(aq)_, CuCO_3(aq)_, NaOH_(aq)_, NaCO_3_^-^_(aq)_, CuCO_3_(OH)_2_^-2^_(aq) _and Cu(CO_3_)_2_^-2^_(aq)_. The calculated Cu adsorbed differed by < 2.5% from those calculated using the more simplified FITEQL model, even for the highest ionic strength experiments. JCHESS was also used to assess saturation states of minerals and to complete speciation calculations for mixed solid assemblages, which is not possible using the FITEQL software. The default JCHESS thermodynamic database contains data for copper-bearing minerals including tenorite, malachite, azurite, and cuprite. No data is included for Cu(OH)_2(s)_. Calculations using the stability constant for formation of Cu(OH)_2(s)_provided with the JCHESS MINTEQ database (log K = -8.64) indicate that for a given set of experimental conditions, tenorite saturates at lower pH then Cu(OH)_2(s)_.

**Table 1:** Surface areas, surface site types and site densities used in DLM calculations.

**Solid**	**Surface Area (m^2^/g)**	**Site Types**	**Site Density (μmol/m^2^)**
HFO (2-site model)	600^[4]^	>Fe_(s)_OH	0.094^[4]^
		>Fe_(w)_OH	3.74^[4]^
HFO (1-site model)	600^[4]^	>FeOH	16.6^[38]^
Kaolinite (KGa)	13.6 (this study)	>SOH	16.6^[38]^
		X	2.2^a^
Kaolinite (Wards)	25.7 (this study)	>SOH	16.6^[38]^
		X	1.2^a^

a. From CEC (3.0 meq/100 g) measured by Bordon and Giese [41] for kaolinite KGA-1b

Adsorption edge data calculated with JCHESS were compared to the experimental data to assess goodness of fit V(Y) using the model proposed by Heinrich et al. [37]:

(1) $$V(Y)=\frac{\sum_{P,Q}{\left(\frac{Y}{s_{Y}}\right)}^{2}}{n_{p}\times n_{Q}-n_{R}}$$

where Y is the difference between the calculated and measured concentration of metal remaining in solution for each data point, P; s_Y _is the standard deviation; n_P _is the number of data points; n_Q _is the number of components, Q, for which the concentration, C, and the total concentration, t, are known (n_Q _= 1 for all edges in this study); and n_R _is the number or parameters being optimized. The standard deviation, s_Y_, was assumed to be equal to 5% of the experimentally measured copper concentration in solution with a lower limit of 10 ppb (based on ICP OES errors and detection limits). Confidence intervals for V(Y) values were calculated according to the equation proposed by Heinrich et al. [37]:

(2) $$\frac{\left(n_{Q}\times n_{P}-n_{R}\right)*V(Y)}{\chi_{1-\alpha /2}^{2}},\frac{\left(n_{Q}\times n_{P}-n_{R}\right)*V(Y)}{\chi_{\alpha /2}^{2}},$$

where *χ*^2^_*p *_is the quantile of the chi-square distribution with (n_Q_xn_P_-n_R_) degrees of freedom with exceedence probability, *p*, and *α *= .05 (95% confidence interval). For a given set of adsorption edges, the simplest model yielding a statistically superior V(Y), i.e. the model with the lowest V(Y) having no overlap with the 95% confidence interval of the next best model, was accepted as the best model. The V(Y), together with the 95% confidence intervals, was also used to compare the fit of models calibrated for the single solids with those obtained for the mixed mineral systems.

## Results and discussion

### Cu adsorption on hydrous ferric oxide (HFO)

**Figure 1:**
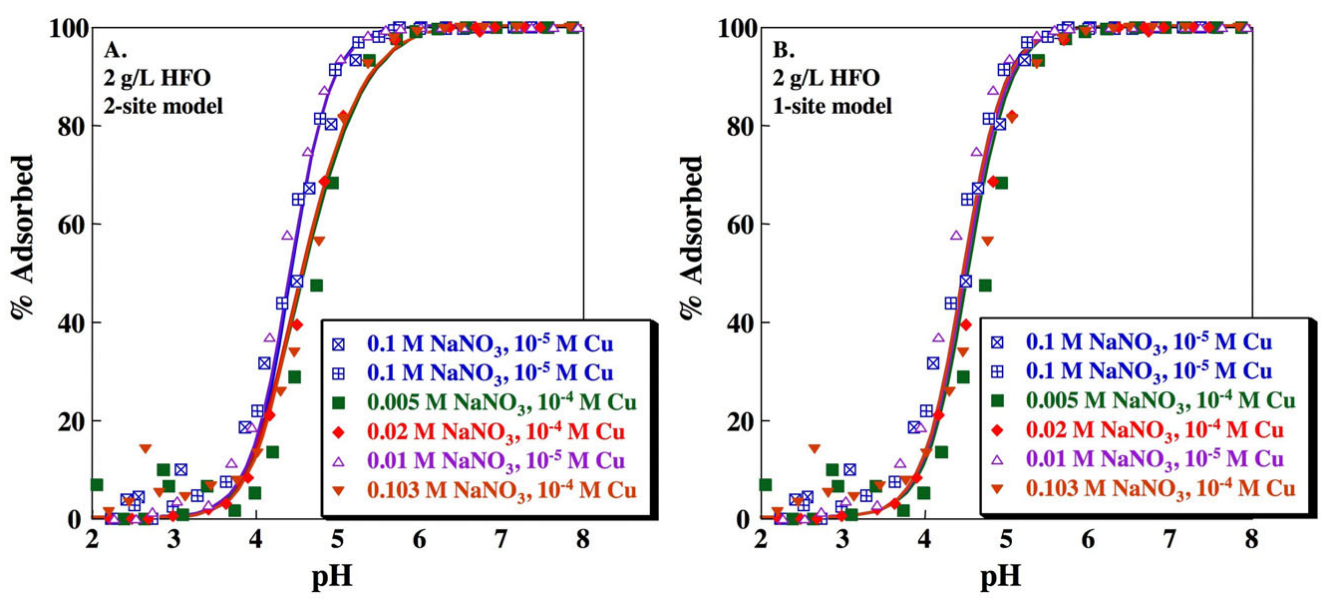
**Cu adsorption as a function of pH on HFO**. Solid concentration is 2 g/L. Lines indicate fits for (A) Dzombak and Morel [4] 2-site HFO model and (B) Sverjensky and Sahai [38] 1-site HFO model calculated using parameters shown in Tables 1 and 2. Replicate experiments (0.1 M NaNO_3 _and 10^-5 ^M Cu) are distinguished by separate symbols.

Cu adsorption on HFO was measured as a function of pH, ionic strength and sorbate/sorbent ratio (Fig. 1). For a given sorbate/sorbent ratio, there is little dependence of adsorption on ionic strength. Increasing the sorbate/sorbent ratio by an order of magnitude, from 10^-5 ^M Cu to 10^-4 ^M Cu on 2 g/L HFO, increases the pH of 50% adsorption (pH_50_) from ~4.4 to ~4.7. Replicate experiments completed with 0.1 M NaNO_3 _and 10^-5 ^M Cu are in reasonable agreement (Fig. 1, blue symbols). JCHESS calculations indicate that the only solid that may become supersaturated in this system is tenorite (CuO). In the absence of adsorption, saturation with respect to tenorite occurs at pH~6 and 6.5, for the 10^-4 ^M and 10^-5 ^M Cu experiments, respectively, which is well above the measured pH edges.

Cu adsorption on HFO has been described by Dzombak and Morel [4] using a 2-site double layer surface complexation model (DLM) with the parameters shown in Tables 1 and 2. Amphoteric strong and weak surface hydroxyl sites are included in the model, but Cu adsorption is assumed to occur as a monodentate complex only on the strong site, according to:

**Table 2:** Reaction stoichiometries and stability constants used in DLM calculations for HFO.

**Reaction**	**Log Stability Constant**	**V(Y) (V(Y)_min_, V(Y)_max_)**
*HFO (2-site model):*		
>Fe_(s)_OH + H^+^_(aq) _= >Fe_(s)_OH_2_^+^	7.29^[4]^	
>Fe_(w)_OH+ H^+^_(aq) _= >Fe_(w)_OH_2_^+^	7.29^[4]^	
>Fe_(s)_OH = >Fe_(s)_O^- ^+ H^+^_(aq)_	-8.93^[4]^	
>Fe_(w)_OH = >Fe_(w)_O^- ^+ H^+^_(aq)_	-8.93^[4]^	14.0
>Fe_(s)_OH + Cu^+2^_(aq) _= >Fe_(s)_OCu^+ ^+ H^+^_(aq)_	2.89^[4]^	(10.9, 18.5)
		
*HFO (1-site model):*		
>FeOH + H^+^_(aq) _= >FeOH_2_^+^	7.5^[38]^	
>FeOH = >FeO^- ^+ H^+^_(aq)_	-10.2^[38]^	12.2
>FeOH + Cu^+2^_(aq) _= >FeOCu^+ ^+ H^+^_(aq)_	0.98 (this study)	(9.5, 16.1)

Average goodness-of-fit parameters (V(Y)) and 95% confidence intervals of V(Y) are for the fit of each model to all of the Cu on HFO adsorption edge data (n_p _= 61).

(3) >Fe_(s)_OH + Cu^+2^_(aq) _= >Fe_(s)_OCu^+ ^+ H^+^_(aq)_.

The resulting model curves are in very good agreement with the experimental data (Fig. 1A; V(Y) = 14.0). The Dzombak and Morel model adequately captures both the lack of ionic strength dependence and the shift in the edges with increased sorbate/sorbent ratio. In experiments with 10^-4 ^M Cu, the relatively small number of strong sites (1.13·10^-4 ^M) are nearly, but not entirely, saturated with sorbed Cu at high pH.

An internally consistent set of single-site DLM parameters for the protonation and deprotonation of a wide variety of solids, including HFO and kaolinite, has been predicted by Sverjensky and Sahai [38] based on Born solvation theory. A goal of the current study is to develop robust DLM descriptions of cation adsorption on environmentally-relevant solids, while minimizing the number of fit parameters. Adsorption of Cu on kaolinite is described in this study (see discussion below) using a DLM based on the work of Sverjensky and Sahai [38]. A second goal of this study is to develop internally-consistent DLMs for Cu adsorption on both HFO and kaolinite. Therefore, the Cu adsorption edges for HFO were used to derive a best-fit stability constant using the site density, protonation and deprotonation values recommended by Sverjensky and Sahai [38] and assuming monodentate adsorption of Cu on the single site, according to:

(4) >FeOH + Cu^+2^_(aq) _= >FeOCu^+ ^+ H^+^_(aq)_.

The V(Y) value for the resulting model (V(Y) = 12.2), while slightly lower than that determined for the 2-site Dzombak and Morel [4] model, is not statistically superior (Table 2). Furthermore, the fits obtained with the single-site model, although statistically inseparable at the 95% confidence interval from those of the 2-site model, fail to capture the dependence of the adsorption edges on sorbate/sorbent ratio (Fig. 1B). In general, we recommend choosing the simplest model, with the least number of fitting parameters, when multiple models produce statistically inseparable results. However, the Dzombak and Morel [4] model has been carefully calibrated with a very large dataset (including many metals besides Cu), and this model is already in widespread use. Therefore, we apply both the Dzombak and Morel 2-site model and the simpler 1-site model developed in this study to predict Cu adsorption for systems containing both HFO and kaolinite (see discussion below).

### Cu adsorption on kaolinite

**Figure 2:**
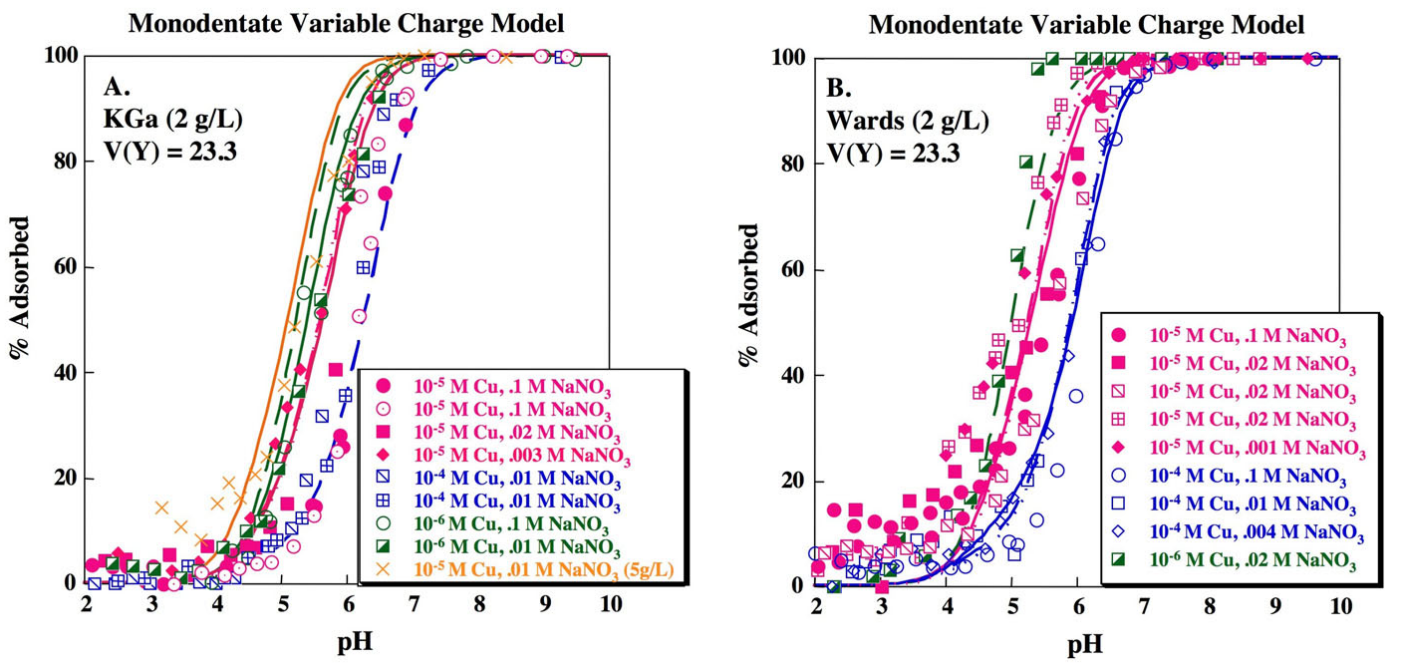
**Cu adsorption as a function of pH on kaolinite**. Solid concentration is 2 g/L unless noted otherwise. Lines indicate fits for 1-site model with formation of a monodentate Cu complex on a variable charge site for (A) KGa and (B) Wards data. Model fits calculated using parameters shown in Tables 1 and 2. Replicate experiments (0.1 M NaNO_3 _and 10^-5 ^M Cu; 0.1 M NaNO_3 _and 10^-4 ^M Cu; 0.02 M NaNO_3 _and 10^-5 ^M Cu;) are distinguished by separate symbols.

Cu adsorption was measured on two types of kaolinite (KGa and Wards) as a function of ionic strength (0.001 to 0.1 M NaNO_3_) and sorbate/sorbent ratio (10^-4 ^to 10^-6 ^M Cu on 2 or 5 g/L kaolinite). The pH_50 _decreases with smaller sorbate/sorbent ratios and typically increases with increasing ionic strength (Fig. 2A, B). This is in agreement with the results of prior studies of Cu adsorption on kaolinite (e.g., [23-28]). Replicate experiments (10^-5 ^M Cu, 0.1 M NaNO_3 _in Fig. 2A; 10^-4 ^M Cu, 0.01 M NaNO_3 _in Fig. 2A; 10^-5 ^M Cu, 0.02 M NaNO_3 _in Fig. 2B) are generally in agreement, although one of the 10^-5 ^M Cu, 0.02 M NaNO_3 _(Fig. 2B) does show higher adsorption then expected. Tenorite is predicted to be supersaturated in the absence of adsorption at pH ≥ 6 for 10^-4 ^M Cu and ≥ 6.5 for 10^-5 ^M Cu experiments. This is well above the 10^-5 ^M Cu sorption edges, but could influence a portion of the 10^-4 ^M Cu edges. However, as discussed above, rapid desorption of 10^-5 ^M Cu from kaolinite after equilibration for 72 hours at pH 10.5 suggests that adsorption is the primary uptake mechanism.

A number of surface complexation models have been proposed to describe Cu adsorption on kaolinite. Most of these follow the lead of Schindler et al. [23] and use a 2-site approach. Schindler et al. [23] derived a 2-site constant capacitance model (CCM) with the formation of an innersphere Cu surface complex on a variable charge site together with exchange of Cu^+2 ^for H^+ ^or Na^+ ^on a permanent charge, or ion exchange site. Schindler et al. [23] interpret the variable charge site as an aluminol site and suggest that the exchange site may either be due to isomorphous substitution giving rise to a small permanent structural charge on the kaolinite or may be due to the presence of a small amount of 2:1 interlayer clay impurity in the kaolinite specimen. Subsequent work by Ikhsan et al. [25] used a similar modeling approach except that a bidentate complex was used to describe Cu adsorption on the variable charge site. Likewise, Heidmann et al. [26] chose a 1-pK Stern model to describe Cu adsorption on variable charge edge sites of kaolinite with ion exchange sites used to describe adsorption at lower pH. An alternative approach for modeling Cu adsorption on kaolinite was proposed by Jung et al. [24], who used a triple layer surface complexation model assuming that Cu binds as an innersphere monodentate complex on an amphoteric aluminol site and as an outersphere monodentate complex on a deprotonatable silanol site. Similarly, Hizal and Apak [28] assumed formation of monodentate Cu complexes on two variable charge sites, presumed to correspond to aluminol and silanol sites. Finally, Peacock and Sherman [27] used an extended constant capacitance model to describe Cu adsorption on kaolinite. Using EXAFS data as a guide, Peacock and Sherman [27] proposed a model with Cu sorption occurring at three sites, forming a bidentate, mononuclear innersphere complex with an aluminol edge site; a tridentate, binuclear complex with an aluminol edge site; and binding to an ion exchange site on the basal plane of the kaolinite.

The goal of this study is to derive a simple, DLM description of Cu binding on kaolinite consistent with the DLM description of Cu binding on HFO that can be used to make predictions of Cu speciation in mixed solid systems. A variety of models were tested, including monodentate or bidentate binding of Cu to an amphoteric variable charge site in the presence or absence of an ion exchange site (see Table 3).

**Table 3:** Reaction stoichiometries and stability constants used in DLM calculations for kaolinite.

**Reaction**	**Log Stability Constant**	**V(Y) (V_min_(Y), V_max_(Y))**
>KaoliniteOH + H^+^_(aq) _= >KaoliniteOH_2_^+^	2.1^[38]^	
>KaoliniteOH = >KaoliniteO^- ^+ H^+^_(aq)_	-8.1^[38]^	
		
*Monodentate variable charge site:*		23.3
>KaoliniteOH + Cu^+2^_(aq) _= >KaoliniteOCu^+ ^+ H^+^_(aq)_	-1.7 (this study)	(20.2, 27.1)
		
*Bidentate variable charge site:*		28.5
2 > KaoliniteOH + Cu^+2^_(aq) _= >KaoliniteO_2_Cu + 2H^+^_(aq)_	-4.6 (this study)	(24.8, 33.2)
		
*Monodentate variable charge + ion exchange site (all log K's fit in this study):*		
>KaoliniteOH + Cu^+2^_(aq) _= >KaoliniteOCu^+ ^+ H^+^_(aq)_	-1.9 (this study)	
X(Na) + H^+^_(aq) _= X(H) + Na^+^_(aq)_	4.1 (this study)	44.9
2X(Na) + Cu^+2^_(aq) _= X_2_(Cu) + 2Na^+^_(aq)_	0.72 (this study)	(38.9, 52.2)
		
*Monodentate variable charge + ion exchange site model (fixed Na-H exchange stability constant):*		
>KaoliniteOH + Cu^+2^_(aq) _= >KaoliniteOCu^+ ^+ H^+^_(aq)_	-2.3 (this study)	
X(Na) + H^+^_(aq) _= X(H) + Na^+^_(aq)_	4.3 (this study)	108
2X(Na) + Cu^+2^_(aq) _= X_2_(Cu) + 2Na^+^_(aq)_	2.5^[7]^	(93.6, 126)
		
*Bidentate variable charge + ion exchange site model (fixed Na-H exchange stability constant):*	-5.3 (this study)	
2>KaoliniteOH + Cu^+2^_(aq) _= >KaoliniteO_2_Cu + 2H^+^_(aq)_	4.6 (this study)	62.6
X(Na) + H^+^_(aq) _= X(H) + Na^+^_(aq)_	2.5^[7]^	(54.4, 72.9)
2X(Na) + Cu^+2^_(aq) _= X_2_(Cu) + 2Na^+^_(aq)_		
		
*Monodentate variable charge + ion exchange site model, no Cu sorption on ion exchange site (fixed Na-H exchange stability constant):*	-1.8 (this study) 2.5^[7]^	190 (165, 221)
>KaoliniteOH + Cu^+2^_(aq) _= >KaoliniteOCu^+ ^+ H^+^_(aq)_		
X(Na) + H^+^_(aq) _= X(H) + Na^+^_(aq)_		
		
*Bidentate variable charge + ion exchange site model, no Cu sorption on ion exchange site (fixed Na-H exchange stability constant):*	-4.6 (this study) 2.5^[7]^	33.8 (29.4, 39.3)
2 > KaoliniteOH + Cu^+2^_(aq) _= >KaoliniteO_2_Cu + 2H^+^_(aq)_		
X(Na) + H^+^_(aq) _= X(H) + Na^+^_(aq)_		

The same protonation/deprotonation constants for the variable charge site is used in all models. Average goodness-of-fit parameters (V(Y)) and 95% confidence intervals of V(Y) are for the fit of each model to all of the Cu on kaolinite adsorption edge data (n_p _= 360).

Variable charge surface site densities can be estimated from crystallographic considerations, although this typically yields a range of values, depending on assumptions made regarding crystal morphology and the definition of a surface site (see discussion in [39]). For example, Koretsky et al. [39] estimated a range of 0 to 21.8 sites/nm^2 ^for kaolinite, based on crystallographic considerations. Due to this uncertainty, site densities are often treated as additional fit parameters in surface complexation models. Previous work demonstrates that surface complexation stability constants are dependent on the choice of site densities and that typically a wide range of site densities can provide a satisfactory fit to measured data (e.g., [40]). Therefore, in this study the variable charge surface site density is set equal to 10 sites/nm^2^, as recommended by Sverjensky and Sahai [38]. For models with an additional ion exchange site, the ion exchange site density was calculated directly from the measured cation exchange capacity for KGA-1b reported by Bordon and Giese [41] (Table 1).

Protonation and deprotonation stability constants are typically fit using measured potentiometric acid-base titrations for the mineral of interest. However, Sverjensky and Sahai [38] have developed a predictive scheme for estimating protonation and deprotonation stability constants for a wide variety of solids, based on Born solvation theory. There are several advantages to using their predicted constants. First, if their method produces satisfactory results, then development of the SCM is simplified, requiring fewer experimental measurements and fewer fit parameters. Secondly, their estimates are produced from analysis of many experimental datasets and therefore should be both robust and internally consistent. Finally, although perhaps difficult, a goal of this study is to develop a generally-applicable model of Cu adsorption on kaolinite, which is not specific to a particular specimen of kaolinite. Therefore, protonation and deprotonation stability constants for the variable charge site were taken from the predictions of Sverjensky and Sahai [38] (Table 3) and were not treated as fit parameters, in spite of the fact that doing so might produce a better model fit to the data.

Fits were assessed by calculating the goodness-of-fit for the median stability constant(s) when applied to all of the measured edge data (n_p _= 360). The lowest V(Y) is obtained for the simplest model tested: a single variable site model with formation of a monodentate Cu complex (Table 2; Fig. 2) according to:

(5) >KaoliniteOH + Cu^+2^_(aq) _= >KaoliniteOCu^+ ^+ H^+^_(aq)_.

**Figure 3:**
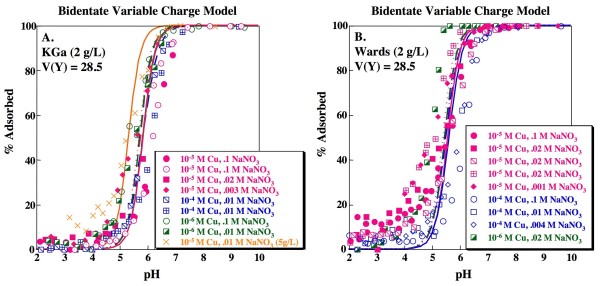
**Cu adsorption as a function of pH on kaolinite**. Solid concentration is 2 g/L unless noted otherwise. Lines indicate fits for 1-site model with formation of a bidentate Cu complex on a variable charge site for (A) KGa and (B) Wards data. Model fits calculated using parameters shown in Tables 1 and 2. Replicate experiments (0.1 M NaNO_3 _and 10^-5 ^M Cu; 0.1 M NaNO_3 _and 10^-4 ^M Cu; 0.02 M NaNO_3 _and 10^-5 ^M Cu;) are distinguished by separate symbols.

This model produces a reasonable description of the pH edge dependence on ionic strength and sorbate/sorbent for both specimens of kaolinite although, especially for the Wards kaolinite, sorption is somewhat underestimated at low pH (< 4.5). XAS and macroscopic isotherm data suggest the formation of a bidentate Cu complex on kaolinite [25,27]. Modeling Cu adsorption on kaolinite using a simple single site model with a bidentate Cu complex, i.e.,

(6) 2 > KaoliniteOH + Cu^+2^_(aq) _= >KaoliniteO_2_Cu + 2H^+^_(aq)_,

produces a slightly higher V(Y) compared to the simple monodentate model, although the fit is not statistically distinct at the 95% confidence interval (Table 3). However, visual inspection of the fits produced by the bidentate model suggests that the predicted edges are systematically steeper than the experimental data and also that the description of sorbate/sorbent dependence is poorer than for the monodentate model (Fig. 3).

As described above, 2-site models have been used to describe Cu adsorption on kaolinite. Furthermore, two-site models are necessary to produce adequate descriptions of weaker ion sorption (e.g. Co and Cd) on kaolinite (e.g., [7,25,42]). The eventual goal of the approach in this study is to develop a relatively simple surface complexation model that can be applied in natural systems containing mixtures of multiple solids and cations. Thus, a 2-site approach, even if it does not produce a statistically superior description of the experimental Cu data, may be necessary to describe metal adsorption in natural systems that contain mixtures of weakly and strongly sorbing ions. Therefore, the simple single variable charge site model was expanded to include a permanent charge, or ion exchange, site, which can bind Na^+ ^or Cu^+2 ^according to

**Figure 4:**
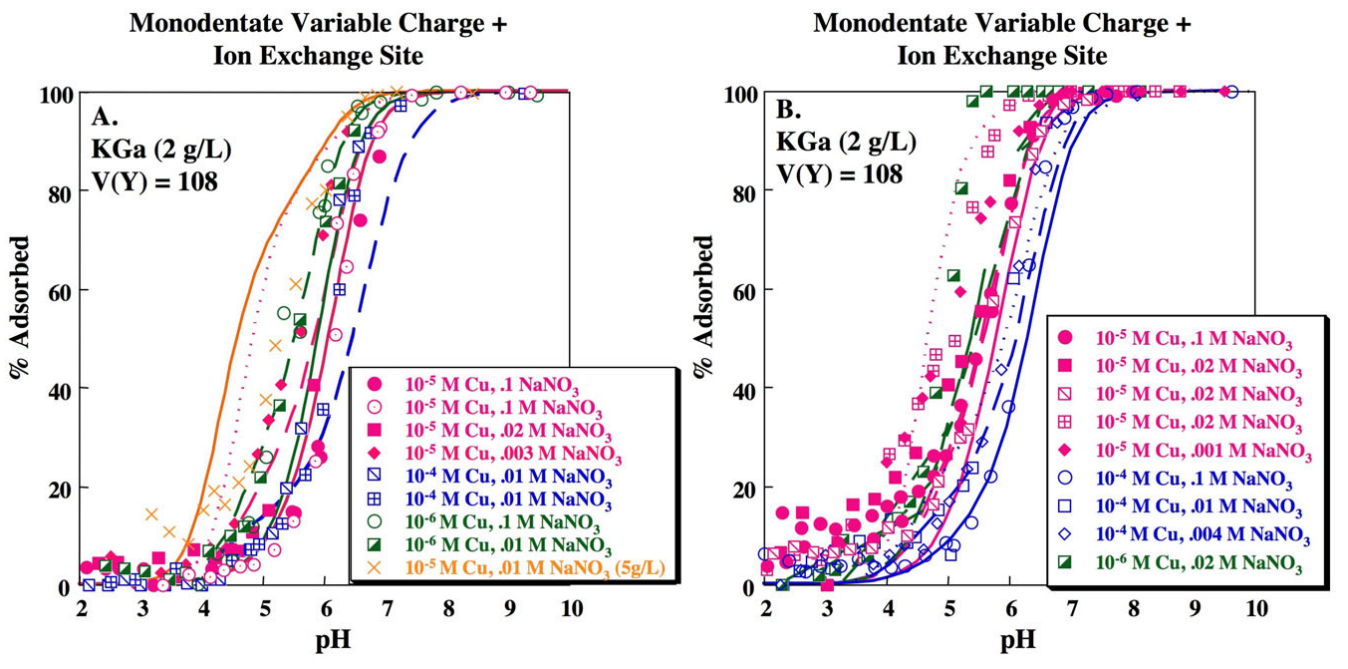
**Cu adsorption as a function of pH on kaolinite**. Solid concentration is 2 g/L unless noted otherwise. Lines indicate fits for 2-site model with formation of a monodentate Cu complex on a variable charge site and Cu sorption on an ion exchange site site (A) KGa and (B) Wards data. Model fits calculated using parameters shown in Tables 1 and 2. Replicate experiments (0.1 M NaNO_3 _and 10^-5 ^M Cu; 0.1 M NaNO_3 _and 10^-4 ^M Cu; 0.02 M NaNO_3 _and 10^-5 ^M Cu;) are distinguished by separate symbols.

**Figure 5:**
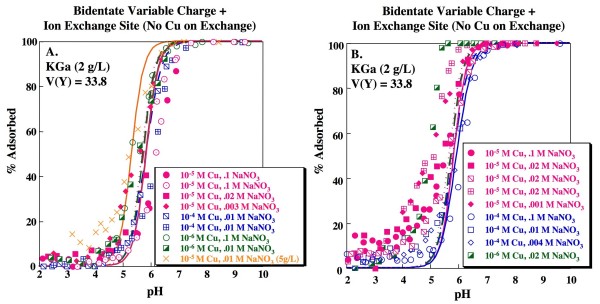
**Cu adsorption as a function of pH on kaolinite**. Solid concentration is 2 g/L unless noted otherwise. Lines indicate fits for 2-site model with formation of a bidentate Cu complex on a variable charge site and an ion exchange site that that does not bind Cu for (A) KGa and (B) Wards data. Model fits calculated using parameters shown in Tables 1 and 2. Replicate experiments (0.1 M NaNO_3 _and 10^-5 ^M Cu; 0.1 M NaNO_3 _and 10^-4 ^M Cu; 0.02 M NaNO_3 _and 10^-5 ^M Cu;) are distinguished by separate symbols.

(7) X(Na) + H^+^_(aq) _= X(H) + Na^+^_(aq) _and

(8) 2X(Na) + Cu^+2^_(aq) _= X_2_(Cu) + 2Na^+^_(aq)_,

respectively (e.g. [7,42]). Although good fits, correctly describing the slightly elevated sorption at low pH, are obtained for individual edges by fitting for the three stability constants associated with reactions (5), (7) and (8), applying the median values to the full set of data results in a statistically poorer fit to the kaolinite data compared to the 1-site model (Table 3). Furthermore, for many experiments, FITEQL did not converge when stability constants for reactions (6), (7) and (8) were fit simultaneously. Landry et al. [7] found that a 2-site approach is necessary to adequately describe Co adsorption on kaolinite. Using an amphoteric variable charge site and an ion exchange site as shown above, Landry et al. [7] derived a stability constant of 2.5 for reaction (7). Using this value for the Na-H exchange stability constant and fitting for the stability constants associated with Cu binding on the ion exchange site and on the variable charge site (as either a bidentate or monodentate complex) still yields a statistically poorer fit to the data compared to the 1-site model (Table 3), producing overestimates of sorption at low ionic strength and sorbate/sorbent ratio and underestimates of sorption at high ionic strength and sorbate/sorbent ratio, particularly on KGa kaolinite (Fig. 4). The best model fit with the ion exchange included is obtained when Cu sorption to the ion exchange site (reaction 8) is excluded from the model (i.e. only reaction 7 is allowed) and Cu forms a bidentate site on the variable charge site (V(Y) = 33.8; Table 3; Fig. 5). The goodness-of-fit for this model is not statistically different from the 1-site bidentate model, although it is not as good as the 1-site monodentate model at the 95% confidence interval. Nonetheless, this model has the advantage that it is consistent with the 2-site models required to describe adsorption of weakly binding ions such as Cd^+2 ^or Co^+2 ^on kaolinite. It is also consistent with formation of a bidentate Cu species as inferred from isotherm data [25] and XAS studies [27].

**Figure 6:**
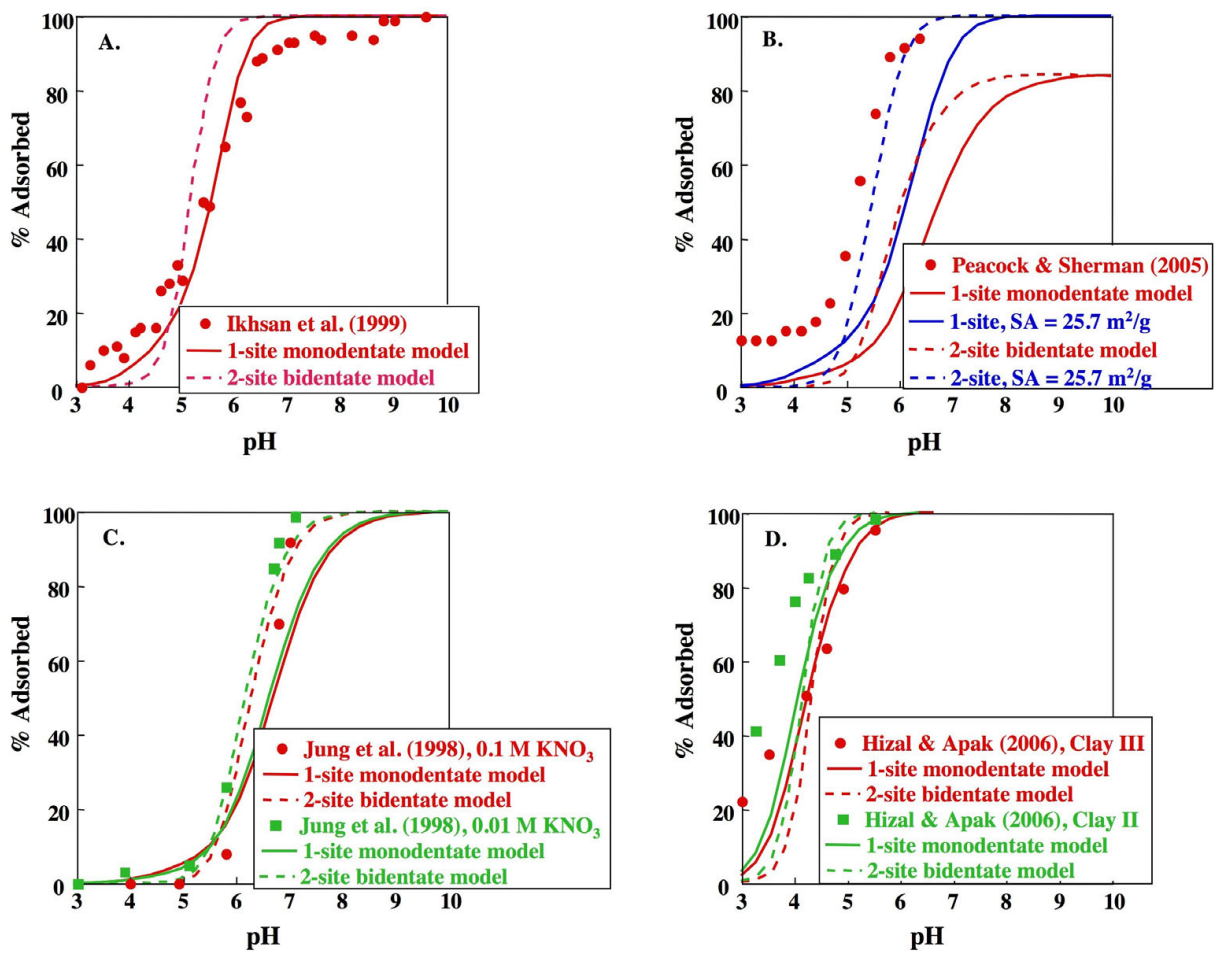
**Comparisons 1-site and 2-site kaolinite models with previously reported data**. The 1-site model includes a monodentate Cu complex on a variable charge site. The 2-site model includes a bidentate cu complex on a variable charge site and an ion exchange site that does not bid Cu (parameters shown in Table 2). Data is from: (A) Ikhsan et al. [25] for 0.005 M KNO_3_, 10^-4 ^M Cu, 6.8 g/L kaolinite with 14.73 m^2^/g surface area, (B) Peacock and Sherman [27] for 0.1 M NaNO_3_, 3.93·10^-4 ^M Cu, 3.33 g/L kaolinite with 12.2 m^2^/g, (C) Jung et al. [24] for 0.01 or 0.1 M KNO_3_, 10^-4 ^M Cu, 2.0 g/L kaolinite with 7.99 m^2^/g and (D) Hizal and Apak [28] with 0.1 M NaClO_4_, 1.57·10^-4 ^M Cu, 50 g/L kaolinite with 26.68 m^2^/g (Clay II) or 17.8 m^2^/g (Clay III).

Predictions from the two best models, i.e., the 1-site model (monodentate Cu adsorption on a variable charge site) and the 2-site model (ion exchange site that does not sorb Cu; variable charge site with bidentate adsorption of Cu) were compared to adsorption edge data obtained in previous studies [24,25,27,28]. For calculations with the 2-site model, the electrolyte was assumed to be NaNO_3_, although two studies used KNO_3 _[24,25] and one used NaClO_4 _[28]. The 1-site monodentate model produced a V(Y) of 634 for all of the compiled data (n_p _= 73), whereas the 2-site bidentate model resulted in a significantly better fit to the complete dataset (V(Y) = 281; Fig. 6). The adsorption edge reported by Ikhsan et al. [25] is in reasonable agreement with predictions from the 1-site monodentate model derived independently in this study (Fig. 6A). The 2-site bidentate model yields a pH_50 _within ~0.3 of the measured pH_50_, but the predicted edge is steeper than the data of Ikhsan et al. [25]. Both models predict saturation of the surface at less than 100% for the conditions reported by Peacock and Sherman ([27]; Fig. 6B). The 2-site bidentate model produces a reasonable fit to both datasets reported by Jung et al. [24], while the 1-site monodentate model underestimates the amount of Cu adsorbed (Fig. 6C). The 1-site mondentate model is in slightly better agreement with the data of Hizal and Apak [28] at high pH compared to the 2-site bidentate model, which produces a steeper edge, but both models underestimate adsorption compared to the data at lower pH (Fig. 6D).

The general agreement between the predicted edges from the model derived here and data from four independent studies using different ionic strength, background electrolyte, sorbate/sorbent ratio and kaolinite specimens is encouraging. Discrepancies between model predictions and these experiments could be due to differences in experimental conditions, for example in the choice of background electrolyte (e.g., KNO_3 _or NaClO_4 _rather than NaNO_3_). Another possibility is that the disagreement reflects differences in the purity, solid solution chemistry, defect structure or other characteristics of the kaolinite specimens. Differences in measured N_2_-BET surface areas of the kaolinite specimens may also play a role in the model misfits. For example, Peacock and Sherman [27] report an N_2 _BET surface area of 12.2 m^2^/g for the Cornwall kaolinite used in their study. This, combined with the site density of 10 sites/nm^2 ^chosen here, results in saturation of the surface at ~80%, and underestimates the percentage of Cu adsorbed reported by Peacock and Sherman [27]. Increasing the surface area to 25.7 m^2^/g (as measured for the Wards sample in this study) results in much better agreement with the reported data (Fig. 6B). The significant dependence of derived stability constants on measured surface area and choice of site density is well known (e.g. [40,43]), and may prove to be particularly problematic in the application of surface complexation models to natural sediments, where reactive surface area is difficult to assess [3].

### Cu adsorption on mixture of kaolinite and hydrous ferric oxide

**Figure 7:**
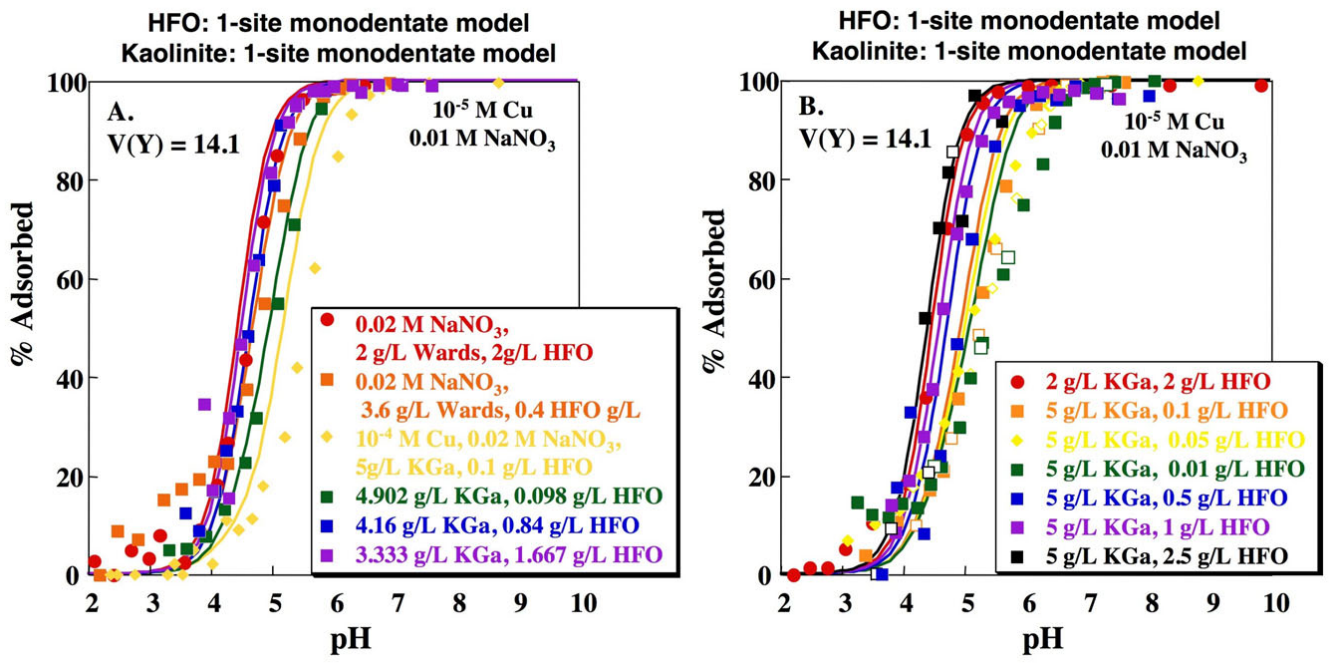
**Predicted Cu adsorption as a function of pH on mixtures of kaolinite and HFO**. Lines indicate fits using the Sverjensky and Sahai [38] 1-site HFO model and 1-site kaolinite model with formation of a monodentate Cu complex on a variable charge site. Model fits calculated using parameters shown in Tables 1, 2 and 3. Open and closed symbols indicate experiments with base titration (closed symbols) followed by acid titration (open symbols).

Cu adsorption was measured on mixtures of HFO and kaolinite (both Wards and KGa) at a range of conditions, although most experiments were conducted in 0.01 M NaNO_3 _and using 10^-5 ^M Cu. Total solid concentrations ranged from 4 g/L to 7.5 g/L and ratios of kaolinite to HFO ranged from 1:1 to 500:1. As might be expected, adsorption increases at a given pH, ionic strength and sorbate/sorbent ratio with increasing quantities of HFO (Fig. 7). Several experiments were conducted with base titration (closed symbols), followed by acid titration (open symbols), to test for hysteresis. Within the experimental uncertainty, no significant hysteresis was observed.

**Figure 8:**
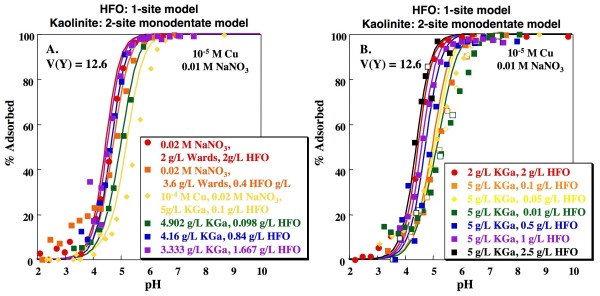
**Predicted Cu adsorption as a function of pH on mixtures of kaolinite and HFO**. Lines indicate fits using the Sverjensky and Sahai [38] 1-site HFO model and 2-site kaolinite model with formation of a monodentate Cu complex on a variable charge site and no Cu adsorption on the ion exchange site. Model fits calculated using parameters shown in Tables 1, 2 and 3. Open and closed symbols indicate experiments with base titration (closed symbols) followed by acid titration (open symbols).

Goodness-of-fit parameters for the mixed solid systems (n_p _= 255) were assessed for various combinations of the single solid models discussed above (Table 4). The resulting V(Y) range from 12.6 to 58.2, with values typically intermediate between those obtained for the pure HFO and pure kaolinite systems. In the pure systems, the 1-site HFO model and the 1-site monodentate variable charge kaolinite model produced the lowest V(Y) values (12.2 and 23.3, respectively). Combining these two models yields V(Y) = 14.1 (Fig. 7). Although this is not the lowest V(Y) obtained by combining individual solid models, it is not statistically distinguishable from the lowest value of 12.6 (Fig. 8), obtained by combining the 1-site HFO model with the 2-site monodentate kaolinite model, without sorption of Cu on the ion exchange site, at the 95% confidence interval.

**Table 4:** Average goodness-of-fit parameters (V(Y)) and 95% confidence intervals of V(Y) for the fit of each model to all of the Cu on kaolinite + HFO adsorption edge data (n_p _= 255). Exchange site models use reaction (7) stability constant of 2.5 from Landry et al. [7]

**Model HFO/Kaolinite**	**V(Y) (V_min_(Y), V_max_(Y))**
*DM/MV*	18.0 (15.2, 21.6)
*DM/BV*	13.6 (11.5, 16.3)
*DM/MVE*	58.2 (49.3, 69.8)
*DM/BVE*	27.0 (22.8, 32.3)
*DM/MVE(noCu)*	21.6 (18.3, 25.9)
***DM/BVE(noCu)***	**13.6 (11.5, 16.3)**
	
*SS/MV*	14.1 (12.0, 16.9)
*SS/BV*	15.9 (13.5, 19.1)
*SS/MVE*	19.2 (16.3, 23.1)
*SS/BVE*	29.1 (24.6, 34.9)
*SS/MVE(noCu)*	12.6 (10.7, 15.1)
*SS/BVE(noCu)*	15.9 (13.5, 19.1)

DM = 2-site HFO model from [4]

SS = 1-site HFO model

MV = 1-site monodentate variable charge kaolinite model

BV = 1-site bidentate variable charge kaolinite model

MVE = 2-site kaolinite model with monodentate variable charge and ion exchange site

MVB = 2-site kaolinite model with bidentate variable charge and ion exchange site

MVE(noCu) = 2-site kaolinite model with monodentate variable charge and ion exchange site (no Cu Sorption on exchange site)

MVBnoCu) = 2-site kaolinite model with bidentate variable charge and ion exchange site (no Cu Sorption on exchange site)

**Figure 9:**
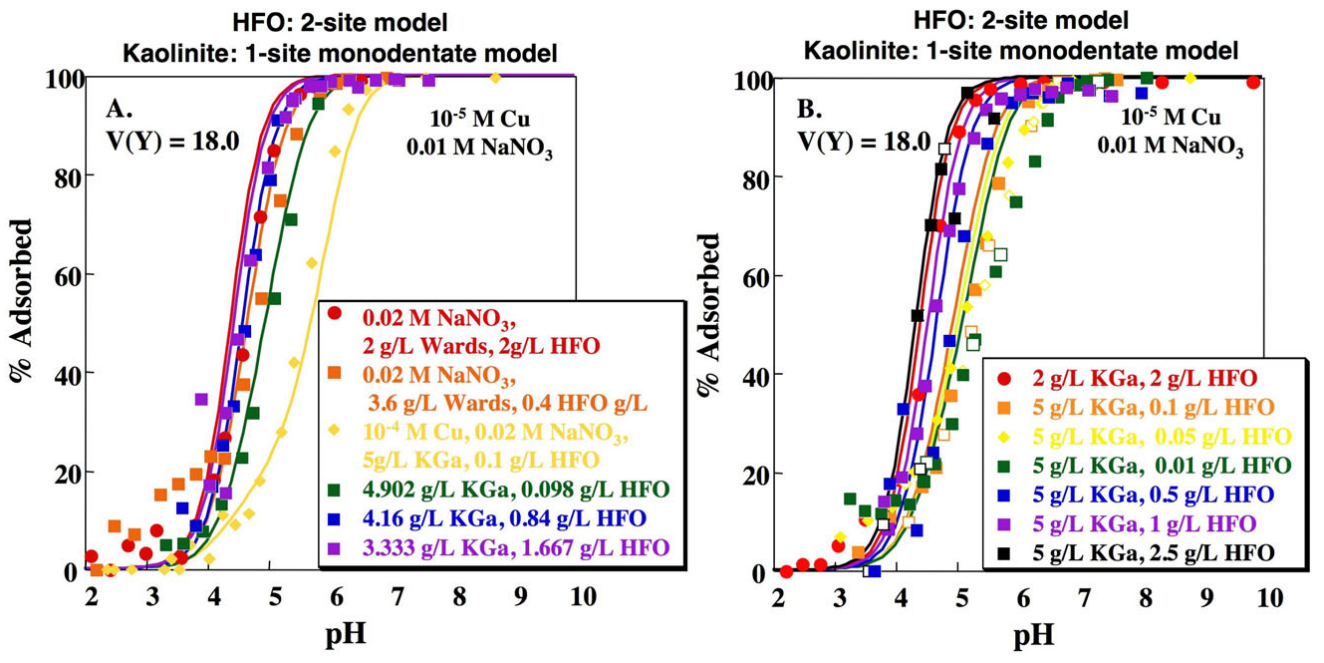
**Predicted Cu adsorption as a function of pH on mixtures of kaolinite and HFO**. Lines indicate fits using the Dzombak and Morel [4] 2-site HFO model and the 1-site kaolinite model with formation of a monodentate Cu complex on a variable charge site. Model fits calculated using parameters shown in Tables 1, 2 and 3. Open and closed symbols indicate experiments with base titration (closed symbols) followed by acid titration (open symbols).

Although combining the simplest models producing statistically indistinguishable V(Y) values for the individual systems is conceptually appealing, these models may not represent the best DLM descriptions of the systems. As discussed above, the 2-site HFO model of Dzombak and Morel [4] produces a visually better fit to the HFO data and has been calibrated for a greater breadth of data than the 1-site HFO model presented above. Combining the 1-site monodentate kaolinite model with the 2-site HFO model results in a V(Y) of 18.0 (Fig. 9). As can be seen in Figs. 7 and 9, combining either HFO model with the 1-site monodentate kaolinite model produces a reasonable fit to the measured edges, although in both cases sorption is overestimated for experiments with the greatest kaolinite to HFO ratio. Use of the 2-site HFO model produces a better prediction of the dependence of sorption on sorbate/sorbent ratio (Figs. 7A, 9A; yellow data/lines).

**Figure 10:**
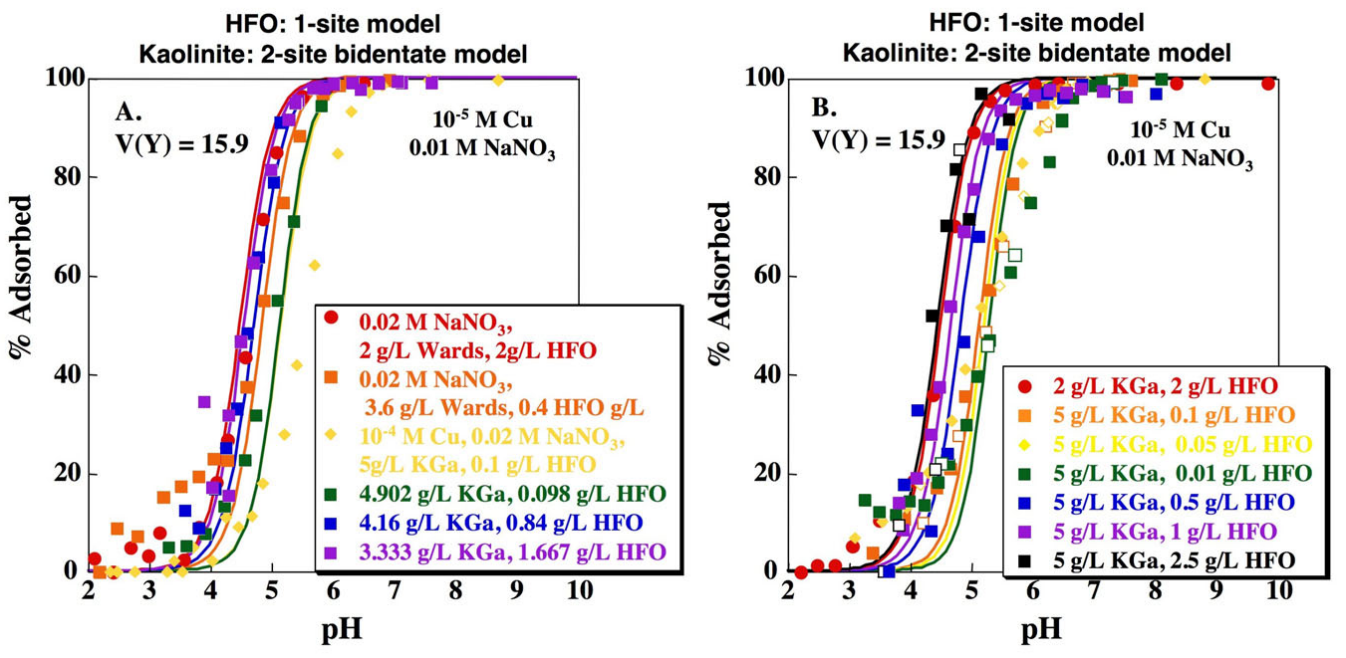
**Predicted Cu adsorption as a function of pH on mixtures of kaolinite and HFO**. Lines indicate fits using the Sverjensky and Sahai [38] 1-site HFO model and the 2-site kaolinite model with formation of a bidentate Cu complex on a variable charge site and an ion exchange site that does not bind Cu. Model fits calculated using parameters shown in Tables 1, 2 and 3. Open and closed symbols indicate experiments with base titration (closed symbols) followed by acid titration (open symbols).

**Figure 11:**
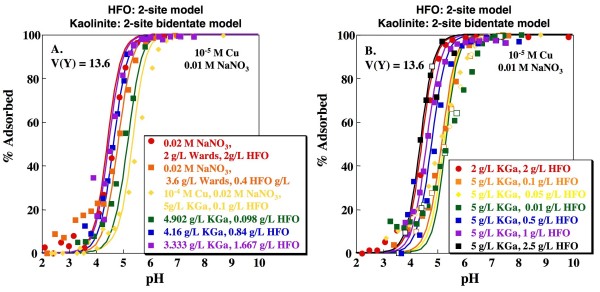
**Predicted Cu adsorption as a function of pH on mixtures of kaolinite and HFO**. Lines indicate fits using the Dzombak and Morel [4] 2-site HFO model and the 2-site kaolinite model with formation of a bidentate Cu complex on a variable charge site and an ion exchange site that does not bind Cu. Model fits calculated using parameters shown in Tables 1, 2 and 3. Open and closed symbols indicate experiments with base titration (closed symbols) followed by acid titration (open symbols).

As discussed above, the 2-site kaolinite model with inclusion of an ion exchange site that does not significantly bind Cu may be preferable to the simpler 1-site model because of its potential for representing adsorption of weaker ions to kaolinite in mixed metal solutions. Combining the 1-site or 2-site HFO models with the 2-site bidentate kaolinite model produces V(Y) values of 15.9 and 13.6, respectively (Figs. 10, 11). The resulting edges are again somewhat steeper than the experimental data, but do adequately capture the dependence of the edges on kaolinite to HFO ratios.

In all cases, surface complexation models calibrated for the single mineral systems do an adequate job of describing metal adsorption in the mixed solid systems. Goodness-of-fit parameters are intermediate between those obtained for the pure HFO and pure kaolinite systems. Discrepancies between model predictions and measured data are similar to those observed in the pure mineral systems. For example, edges that are too steep are produced from kaolinite models assuming bidentate adsorption of Cu in both the pure and mixed mineral systems. Similarly, the 2-site HFO model produces better descriptions of the dependence of Cu sorption on sorbate/sorbent ratio in both pure and mixed mineral systems. The relatively good fits (low V(Y)) obtained for predicted Cu sorption in mixed mineral systems suggest that the stability constants obtained for the pure mineral systems are reasonably robust, and even more importantly, that HFO-kaolinite interactions need not be explicitly included in the speciation model. In other words, a simple component additivity approach produces predictions for the mixed mineral systems that are as good as those for the calibrated pure mineral systems. However, it is important to point out that these experiments were conducted over a relatively brief temporal scale (days). It is possible that aging over months or years may produce surface coatings that physically or chemically block ion adsorption sites. This must be tested using further experimental work, together with adsorption studies on natural sediments and soils.

## Conclusion

This study demonstrates that a simple diffuse layer surface complexation model produces reasonable descriptions of Cu adsorption on HFO and kaolinite over a range of pH, ionic strength, and sorbate/sorbent ratios. In particular, Cu adsorption on kaolinite can be adequately modeled using a very simple DLM with formation of a monodentate Cu complex on a single amphoteric variable-charge site. However, if this model approach is to be extended to natural systems, which contain many other cations, a 2-site approach may be required. Thus, a 2-site kaolinite model, including both a permanent charge and a variable charge site, is developed.

Cu adsorption on mixtures of HFO and kaolinite is predicted reasonably well using a simple component additivity approach, with the DLM parameters derived for the pure mineral systems. Goodness-of-fit values derived for the predicted fits, together with visual inspection of the predictions, suggests that discrepancies between models and data for the mixed mineral systems are similar to those observed for the pure mineral systems. Thus, interactions between the kaolinite and the HFO, such as blocking of ion exchange or variable charge sites, can be neglected in the speciation calculations. However, it is important to note that all of the experiments in this study were conducted over short temporal scales, and that aging could produce coatings or other physical changes not apparent in the present study. Further experiments must be completed using longer timescales to assess the possible effects of aging on Cu speciation in mixed solid systems. Nonetheless, the results of this study are encouraging, suggesting that relatively simple models with few adjustable parameters may produce useful predictions of metal speciation in natural sediments and soils containing many solid components. However, it is also important to note that testing the component additivity approach for simple systems such as this is only a first step toward application of the component additivity approach to field settings. Even if mineral-mineral interactions can be ignored, methods must still be developed to assess reactive surface areas in field settings if the component additivity approach is to gain widespread use.

## Competing interests

The authors declare that they have no competing interests.

## Authors' contributions

All authors contributed to the design of the experiments and the modeling approach. TJL and MSS carried out all experiments. CMK conceived the study, participated in the design and coordination of all experiments and, together with TJL, drafted the manuscript. All authors read and approved the final manuscript.

## References

[B1] Stumm W, Morgan JJ (1996). Aquatic Chemistry: Chemical Equilibria and Rates in Natural Waters.

[B2] Bethke CM, Brady PV (2000). How the K_d _approach undermines ground water cleanup. Ground Water.

[B3] Koretsky C (2000). The significance of surface complexation reactions in hyrologic systems: a geochemist's perspective. Journal of Hydrology.

[B4] Dzombak DA, Morel F (1990). Surface Complexation Modeling: Hydrous Ferric Oxide.

[B5] Sposito G (1984). The Surface Chemistry of Soils.

[B6] Spathariotis E, Kallianou C (2007). Adsorption of copper, zinc, and cadmium on goethite, aluminum-substituted goethite, and a system of kaolinite-goethite: surface complexation modeling. Communications in Soil Science Plant Anal.

[B7] Landry CJ, Koretsky CM, Lund TJ, Schaller M, Das S Surface complexation modeling of Co(II) adsorption on mixtures of hydrous ferric oxide, silica and kaolinite. Geochimica et Cosmochimica Acta.

[B8] Yu S, He ZL, Huang CY, Chen GC, Calvert DV (2002). Adsorption-desorption behavior of copper at contaminated levels in red soils from China. Journal of Environmental Quality.

[B9] McBride MB, Martinez CE (2000). Copper phytotoxicity in a contaminated soil: remediation tests with adsorptive materials. Environmental Science & Technology.

[B10] McBride MB, Martinez CE, Topp E, Evans L (2000). Trace metal solubility and speciation in a calcareous soil 18 years after no-till sludge application. Soil Science.

[B11] Singh SP, Ma LQ, Tack FMG, Verloo MG (2000). Trace metal leachability of land-disposed dredged sediments. Journal of Environmental Quality.

[B12] Sukreeyapongse O, Holm PE, Strobel BW, Panichsakpatana S, Magid J, Hansen HCB (2002). pH-dependent release of cadmium, copper, and lead from natural and sludge-amended soils. Journal of Environmental Quality.

[B13] Vulkan R, Mingelgrin U, Ben-Asher J, Frenkel H (2002). Copper and zinc speciation of a soil and sludge mixture. Journal of Environmental Quality.

[B14] Wu J, Laird DA, Thompson ML (1999). Sorption and desorption of copper on soil clay components. Journal of Environmental Quality.

[B15] Romkens PFAM, Salomens W (1998). Cd, Cu, and Zn solubility in arable and forest soils: consequences of land use changes for metal mobility and risk assessment. Soil Science.

[B16] Zehetner F, Wenzel WW (2000). Nickel and copper sorption in acid forest soils. Soil Science.

[B17] Buerge-Weirich D, Harl R, Xue H, Behra P, Sigg L (2002). Adsorption of Cu, Cd and Ni on goethite in the presence of natural groundwater ligands. Environmental Science and Technology.

[B18] Maqueda C, Morillo E, Undabeytia T (2002). Co sorption of glyphosate and copper (II) on goethite. Soil Science.

[B19] Ali MA, Dzombak DD (1996). Effects of simple organic acids on sorption of Cu^2+ ^and Ca^2+ ^on goethite. Geochimica et Cosmochimica Acta.

[B20] Richter A, Brendler V, Nebelung C (2005). Blind prediction of Cu(II) sorption onto goethite: current capabilities of diffuse double layer model. Geochimica et Cosmochimica Acta.

[B21] Christl I, Kretzschmar R (1999). Competitive sorption of copper and lead at the oxide-water interface: implications for surface site density. Geochimica et Cosmochimica Acta.

[B22] Dubbin WE, Sposito G, Zavarin M (2000). X-ray absorption spectroscopic study of Cu-glyphosate adsorbed by microcrystalline gibbsite. Soil Science.

[B23] Schindler PW, Liechti P, Westall JC (1987). Adsorption of copper, cadmium, and lead from aqueous solution to the kaolinite/water interface. Netherlands Journal of Agricultural Science.

[B24] Jung J, Cho Y-H, Hahn P (1998). Comparative study of Cu^2+ ^adsorption on goethite, hematite and kaolinite: mechanistic modeling approach. Bulletin Korean Chemical Society.

[B25] Ikhsan J, Johnson BB, Wells JD (1999). A comparative study of the adsorption of transition metals on kaolinite. Journal of Colloid and Interface Science.

[B26] Heidmann I, Christl I, Leu C, Kretzschmar R (2005). Competitive sorption of protons and metal cations onto kaolinite: experiments and modeling. Journal of Colloid and Interface Science.

[B27] Peacock CL, Sherman DM (2005). Surface complexation model for multisite adsorption of copper(II) on kaolinite. Geochimica et Cosmochimica Acta.

[B28] Hizal J, Apak R (2006). Modeling of copper(II) and lead(II) adsorption on kaolinite-based clay minerals individually and in the presence of humic acid. Journal of Colloid and Interface Science.

[B29] Tonkin JW, Balistrieri LS, Murray JW (2004). Modeling sorption of divalent metal cations on hydrous manganese oxide using the diffuse double layer model. Applied Geochemistry.

[B30] Spathariotis E, Kallianou C (2001). Adsorption of copper, zinc, and cadmium on clay fraction of two acid soils: surface complexation modeling. Communications in Soil Science Plant Anal.

[B31] Papini MP, Saurini T, Bianchi A, Majone M, Beccari M (2004). Modeling the competitive adsorption of Pb, Cu, Cd, and Ni onto a natural heterogeneous sorbent material (Italian "red soil). Industrial Engineering Chemical Research.

[B32] Pruett RJ, Webb HL (1993). Sampling and analysis of KGa-1B well-crystallized kaolin source clay. Clays and Clay Minerals.

[B33] Payne TE, Davis JA, Lumpkin GR, Chisari R, Waite TD (2004). Surface complexation model of uranyl sorption on Georgia kaolinite. Applied Clay Science.

[B34] Schwertmann U, Cornell RM (2000). Iron Oxides in the Laboratory: Preparation and Characterization.

[B35] Herbelin AL, Westall J (1999). FITEQL – A computer program for determination of chemical equilibrium constants from experimental data. Dept of Chemistry Rep 99-01.

[B36] Lee J Van der, De Windt L (2000). CHESS Tutorial and Cookbook. User's Guide Nr. LHM/RD/99/05.

[B37] Heinrich HTM, Bremer PJ, McQuillan AJ, Daughney CJ Modelling of the acid-base properties of two thermophilic bacteria at different growth times. Geochimica et Cosmochimica Acta.

[B38] Sverjensky DA, Sahai N (1996). Theoretical prediction of single-site surface-protonation equilibrium constant for oxides and silicates in water. Geochimica et Cosmochimica Acta.

[B39] Koretsky CM, Sverjensky DA, Sahai N (1998). A model of surface site types on oxide and silicate minerals based on crystal chemistry: implications for site types and densities, multi-site adsorption, surface infrared spectroscopy and dissolution kinetics. American Journal of Science.

[B40] Hayes KF, Redden G, Ela W, Leckie JO (1991). Surface complexation models: an evaluation of model parameter estimation using FITEQL and oxide mineral titration data. Journal of Colloid and Interface Science.

[B41] Bordon D, Giese RF (2001). Baseline studies of the clay minerals society source clays: cation exchange capacity measurements by the ammonia-electrode method. Clays and Clay Minerals.

[B42] Schaller M, Koretsky CM, Lund TJ, Landry CJ Adsorption of Cd on mixtures of HFO, kaolinite and silica: a surface complexation modeling approach.

[B43] Sverjensky DA (2003). Standard states for the activities of mineral surface sites and species. Geochimica et Cosmochimica Acta.

